# Estimation of Equivalent Series Resistance (ESR) of Supercapacitors Using Charging/Discharging Kinetics

**DOI:** 10.1002/open.202400444

**Published:** 2025-01-20

**Authors:** Mohamed Y. Adel, Omar B. Mohammed, Ahmad T. Younis

**Affiliations:** ^1^ Electronics Engineering College Ninevah University North east side of Mosul University – Stadium Entrance Gate – Mosul University Mosul Iraq; ^2^ Electronics Engineering College Ninevah University North east side of Mosul University – Stadium Entrance Gate – Mosul University Mosul Iraq

**Keywords:** Supercapacitor, ESR, Charging kinetics, Cell aging

## Abstract

One of the key parameters that affects efficiency, power density and performance of a supercapacitor (SC) is the equivalent series resistance (ESR). In this study we propose a method to estimate ESR from the charging kinetics which has practical applications. Therefore, to study the ESR of the SC we must look at the different factors that affect this resistance. In this study, the rise time extracted from the charging kinetics curves of the SC was used as an indirect method to investigate the ESR of the SC. Three different parameters were taken into account: ionic mobility, solvent material and pore cavity size of the porous electrode. The results offer enlightening information for optimizing the design of the SC with higher performance and smaller ESR values that will be translated into a better energy storage technologies.

## Graphical abstract

This work presents a new method to estimate equivalent series resistance (ESR) of supercapacitor from the charging kinetics. Estimation of ESR can be used to monitor the health of supercapacitors. This method is used to examine the effects of ion mobility, solvent material, and aging on supercapacitor performance. Other methods, like EIS are complex and Costlier than this method.

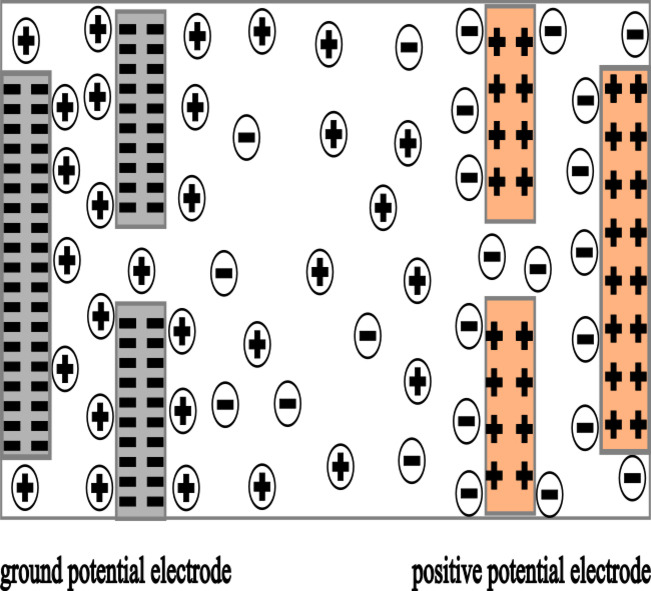

## Introduction

Supercapacitors (SCs), also called electrochemical capacitors, have been widely researched as the next‐generation energy storage system for wearable electronics and electric vehicles owing to their excellent characteristics, such as high power density, long cycle life, fast charge and discharge, and low cost.^[1]^ One of the most important factors affecting SC efficiency and performance is its equivalent series resistor (ESR). The electrolyte resistance, active material resistance and the interface resistance between the active material and current collector are all contributing to the total resistance in a SC circuit which is represented by the ESR.^[2]^ The link between heat generation resistance and current in a SC is made clear by Joules law. In a SC, the charging/discharging current and the ESR have the greatest effect on heat generation according to Joules law.^[3]^ A decrease in capacitance and an increase in ESR are commonly observed during the aging process of a SC.^[4]^ It is imperative to keep an eye on the ESR when assessing the health and efficiency of SCs since variations in this measure may point to malfunctions or internal degradation.^[5]^ Considering that the current flows through all the parts of the SC including electrolyte, electrodes and separator, it is this sum total resistance of the current flow will constitute ESR. Electrochemical impedance spectroscopy (EIS) is used to determine the ESR of a SC experimentally, and shows the impedance signature as Nyquist plot. The Nyquist plot crossing the real axis at the high frequency end is that of ESR value, which is a crucial parameter as it basically will define how our SC will behave.[^6^, ^7^] A high ESR can lead to significant energy losses during the charge/discharge cycles, which in turn compromises the total efficiency of the SC.[^8^, ^9^] While EIS method requires elaborate, expensive pieces of equipment, our method of using charging/discharging kinetics offers the same information about ESR almost effortlessly.

**Figure 1:**
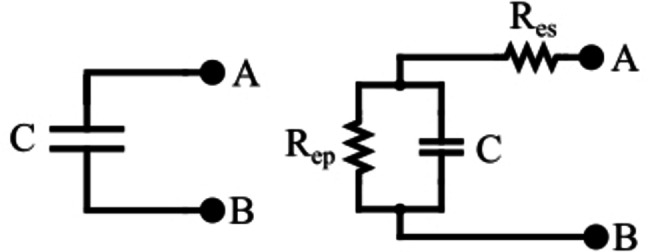
The SC's equivalent circuit.

The equivalent circuit of the SC is given (Figure 1). The elements of the circuit include an ESR (R_es_) representing charge and discharge losses, an equivalent parallel resistance (R_ep_), which simulates the self‐discharge leakage current, and capacitance (C).^[10]^

**Figure 2:**
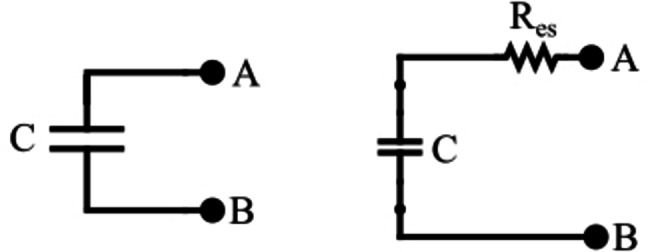
Simplified version of the SC's equivalent circuit.

R_ep_ has a very high resistance value that we can safely neglect it for this study (the leakage current is low compared to charge/discharge currents). The equivalent circuit becomes (Figure 2).^[10]^

ESR is related to power density and charge‐discharge rate capability for the SC. Several strategies have been used to reduce the ESR, which derives from the contributions of electrolyte, electrode resistance and charge transfer resistance. One of the key aspects to minimizing ESR is to improve the electrode materials. For example, using the reduced graphene oxide (rGO) was demonstrated to reduce the ESR by improving the interface between current collector and active material. This is important for SCs because it increases conductor efficiency and reduces contact resistance.^[11]^

## Results and Discussion

### ESR Estimation from Charging Dynamics

In this paper the ESR will be estimated from the charging kinetics concept, as this charging kinetics will provide the time required for the charges to fully accumulate around the porous electrodes of the SC. This charging time along with the capacitance values obtained from the simulated cases will be used to calculate the ESR, using the following equations:

$$\tau =\;t_{r}/(ln(9))$$

where τ represents the time constant, t_r_ represents the rising time, by utilizing the time constant, it is possible to determine the ESR as follows:

R=τ/C

where R is the equivalent series resistance (ESR), C is the capacitance.

### Model Unit Cell

**Figure 3:**
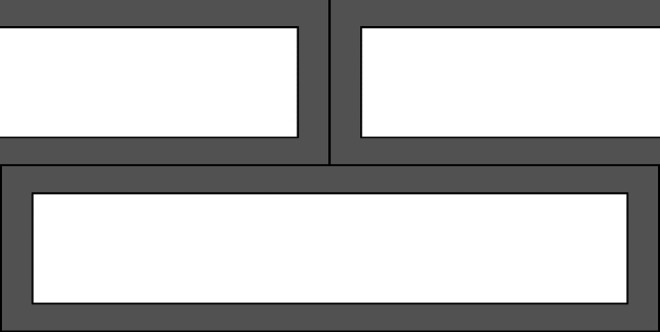
rGO‐ANF structure modeled in COMSOL Multiphysics.

COMSOL Multiphysics was utilized in this study as a simulation tool. The morphology of the porous electrode is based on the scanning electron microscope (SEM) images taken for the reduced graphene oxide with aramid nanofiber layer structure (rGO‐ANF) material.^[12]^ The structure and dimensions of the model based on that images is shown (Figure 3).

“Reprinted with permission from { S. Aderyani, P. Flouda, S. A. Shah, M. J. Green, J. L. Lutkenhaus, and H. Ardebili, “Simulation of cyclic voltammetry in structural supercapacitors with pseudocapacitance behavior,” Electrochimica Acta, vol. 390, p. 138822, Sep. 2021, doi: 10.1016/j.electacta.2021.138822.}. Copyright {2020} American Chemical Society.”

**Figure 4:**
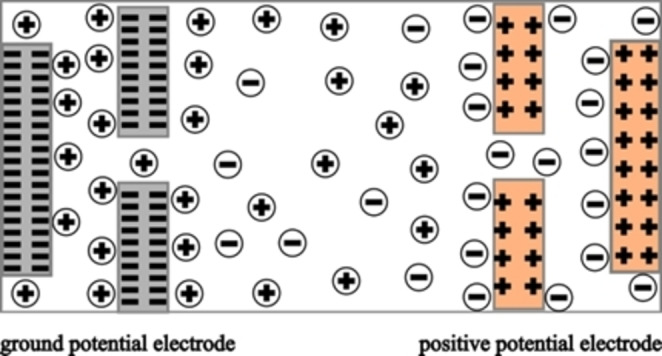
Illustration of the two electrodes SC model.

The complete model and geometry of the SC used for this study is shown (Figure 4). There are one electrode on each side of the channel region which contains the electrolyte with equal quantities of positively and negatively charged ions. At each electrode‐electrolyte interface, an EDL was formed by the ions in motion and the applied potential difference on the electrodes.

**Figure 5:**
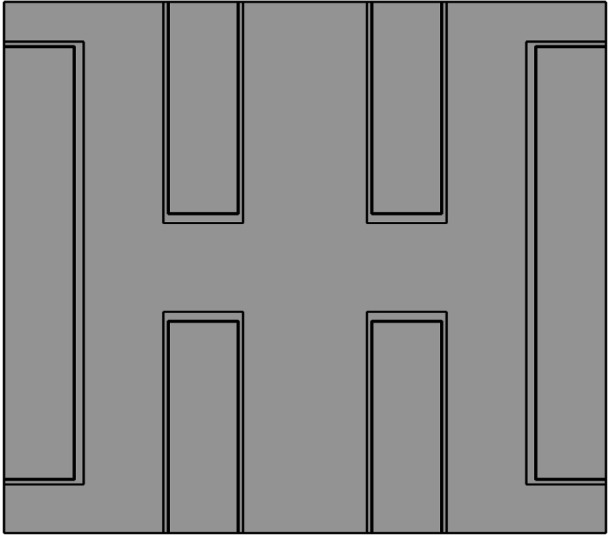
Unit cell of the proposed model.

The schematic of the unit cell of the SC with EDL's Gouy‐Chapman diffused model is shown (Figure 5). The equation of motion that describes how ions move in time through this system is called the Nernst‐Planck equation, which links the applied potential and ions migration (movement under the influence of the field), and get timescales of charging kinetics of an EDL in order to reach equilibrium condition (which is defined by a complete charged EDL).

The Nernst‐Planck equation is:

$${\vec{J}}_{i}=\;-D_{i}\vec{\nabla }c_{i}-\;\left(\frac{z_{i}F}{RT}\right)\;D_{i}c_{i}\vec{\nabla }\varphi$$

where ${\vec{J}}_{i}$ is the molar flux (mol m^−2^ s^−1^), $D_{i}$ is diffusion coefficient of the species i , $c_{i}$ and $z_{i}$ are concentration (mol m^−3^) and the charge number of the species i , respectively. F=96485 (C mol^−1^) is the Faraday's constant, R is the universal gas constant (8.314 J mol^−1^ K^−1^) and T is the temperature. Ion molar flux for species i is related to its concentration and electric potential at any given position in the domain. The equation above is the sum of two terms due to the two fundamental transport processes through which ions are transported across the regions of the SC, the diffusion term, and the migration term.

### The Effect of Ion Species on Charging Kinetics

Each ion species has its own ionic mobility, and to study the effect of ionic species on charging kinetics, a few ion species were used in this study, sodium ion (Na+), silver ion (Ag+) and potassium ion (K+), Table 1 summarizes the mobility of these ions in an aqueous solution.[^13^, ^14^, ^15^]

**Table 1:** Ionic mobility of three different ion species.

Ion species	Ionic mobility^*^
Na+	5.19
Ag+	6.4
K+	7.6
	

*(10^−8^ m^2^ s^−1^ V^−1^).

**Figure 6:**
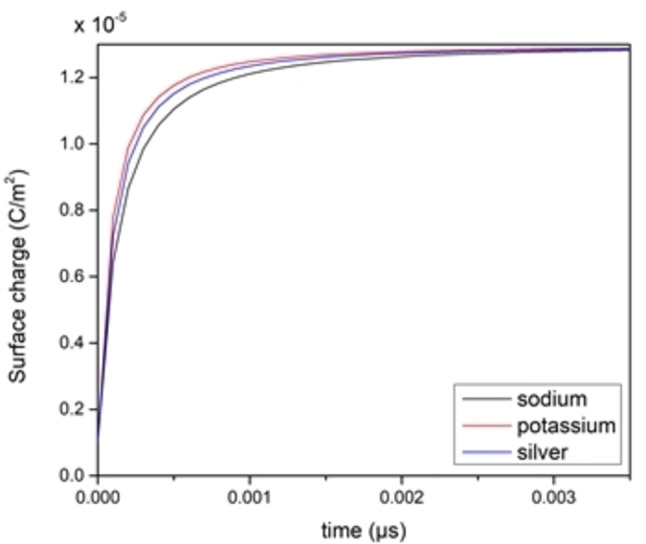
Electrode charge density curves for different ionic species simulations.

**Figure 7:**
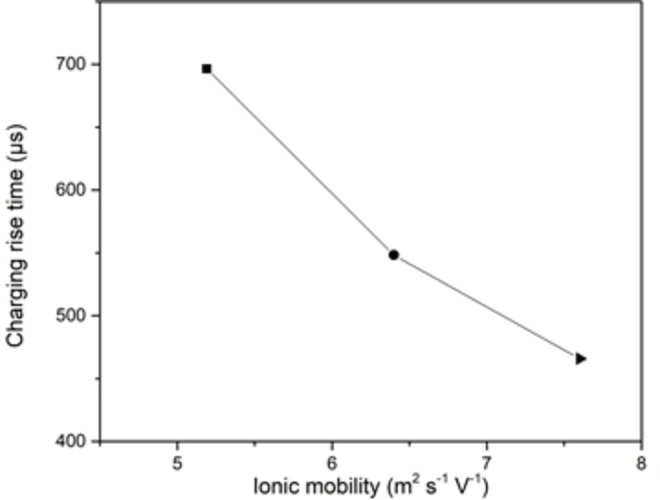
Rise time vs ionic mobility plot for three different ion species.

The time‐varying accumulated charge density for three different ion species is shown (Figure 6). It can be observed that when the mobility of ion species goes up, it allows for charges to build around EDL much quicker. The extracted rising time from the accumulated charge density plots shown (Figure 7) (given that capacitance values extracted from the same plots all have the same value since the accumulated charges around the electrodes all have the same amount, and the applied potential is the same throughout the simulation), show an inverse proportionality with the ionic species mobility which fitted well with the expected behavior, considering that lower ionic radii of ion species in aqueous solution will be surrounded by more of water molecules which results in reducing their ability to move around, thus reducing its mobility according to equation below^[13]^:

μ=ez/6πηr

where μ is the ionic mobility [m^2^ s^−1^ V^−1^], e is elementary charge [1.6×10^19^ C], z is number of ion charges, η is viscosity [N s m^−2^] and r is ionic radius inside the electrolyte.

**Figure 8:**
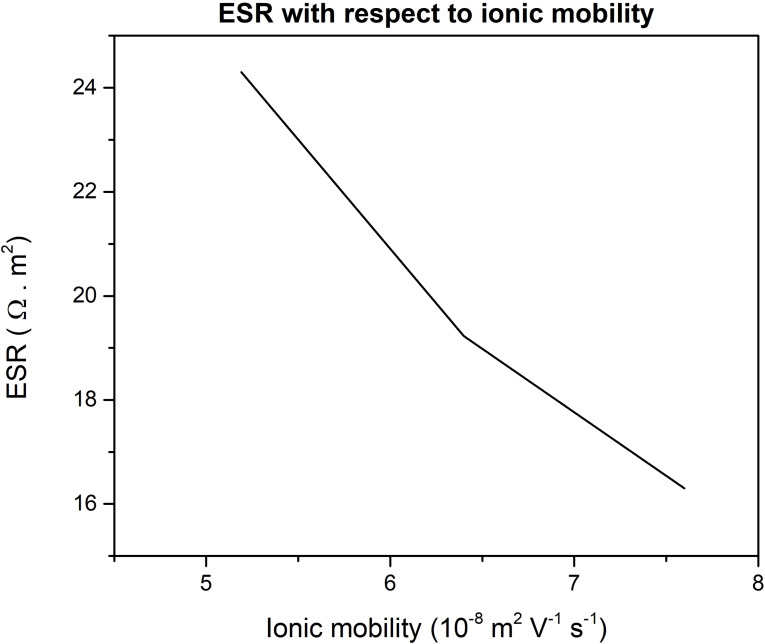
ESR vs ionic mobility plot for three different ion species.

From the curve (Figure 7), it was possible to determine the ESR for the three cases of ion species. The ESR was calculated to be 24.3 Ω m^2^, 19.23 Ω m^2^ and 16.3 Ω m^2^ for sodium, silver, and potassium respectively (Figure 8).

### The Effect of Solvent Material on Charging Kinetics

The ion species carrying the electric charges combined with the solvent in which they get dissolved and move is called an electrolyte. Each solvent material has its own relative permittivity (which affects the SC), and to capture the effect of solvent material on charging kinetics, different solvent materials were used. Table 2 summarizes the materials we used for this study along with its relative permittivity.^[16]^

**Table 2:** Relative permittivity for the materials of the study.

Material	Relative permittivity^*^
Water	80
Methanol	33
Acetone	19.5
	

*Epsilon.

**Figure 9:**
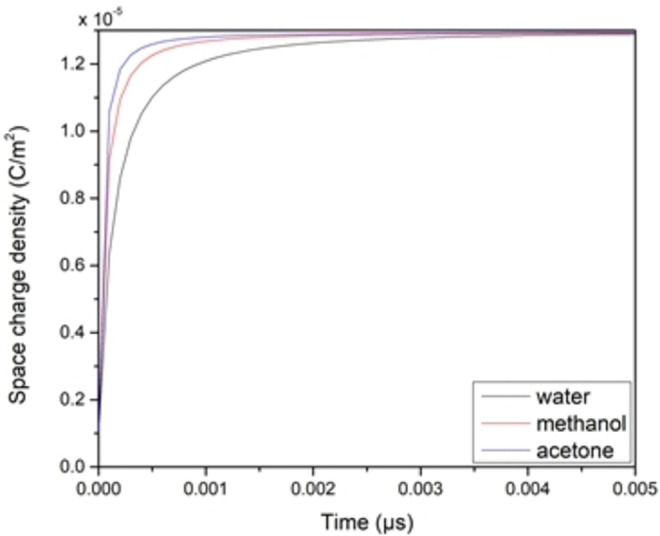
Charging kinetics of various solution substances.

**Figure 10:**
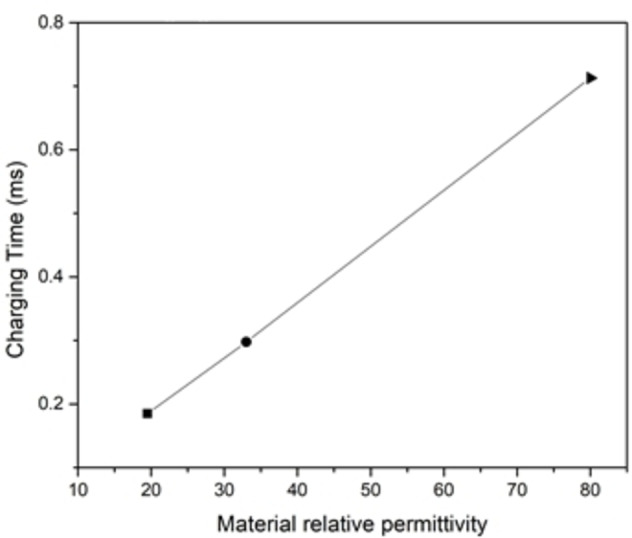
Charging time vs material relative permittivity.

The space charge density around the electrodes as a function of time (charging kinetics) for the three solution materials is shown (Figure 9). The extracted rising time values associated with each solvent material is shown (Figure 10).

It is noted from the figure that the rising time is directly proportional to the relative permittivity of the solvent, given that capacitance values extracted from the same plots all have the same value since the accumulated charges around the electrodes all have the same amount, and the applied potential is the same throughout the simulation, which means that the ESR increased with the increase of the solvent permittivity.

While there is not a direct relationship between ESR and relative permittivity, the choice of electrolyte (which determines ϵ) can indirectly influence ESR by affecting the ionic conductivity. What is of importance with supercapacitors is their ability to absorb and release electric charges, this depends on their ESR, the more conductive the electrolyte the lower is the ESR and consequently the faster the charge and discharge time.[^17^, ^18^]

**Figure 11:**
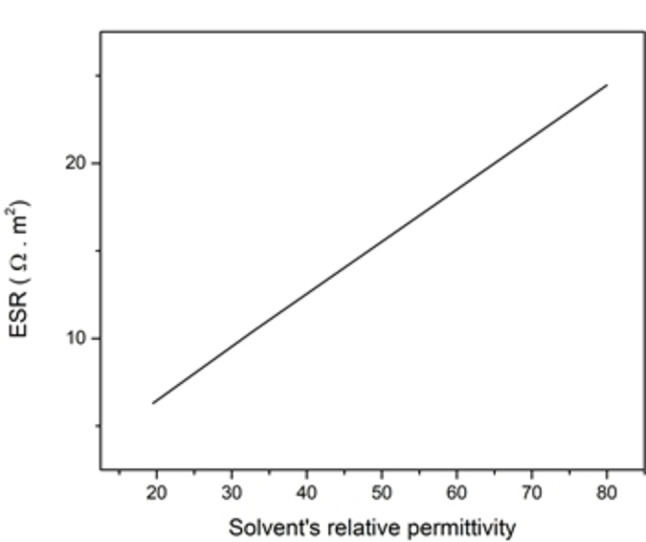
ESR for different solvent materials.

As earlier, the ESR was determined for every case of material's relative permittivity, the ESR values were 24.46 Ω m^2^, 10.46 Ω m^2^, and 6.3 Ω m^2^ for water, methanol, and acetone respectively (Figure 11).

### The Effect of Cell Aging on Charging Kinetics

**Figure 12:**
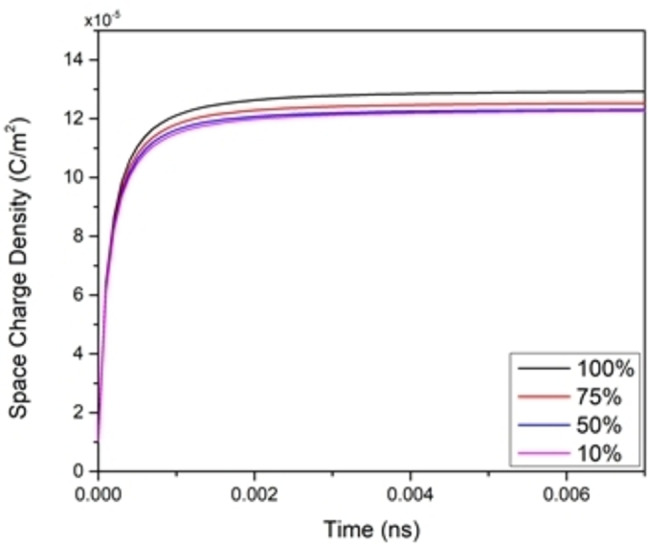
Charge density curve for various pore dimensions.

**Figure 13:**
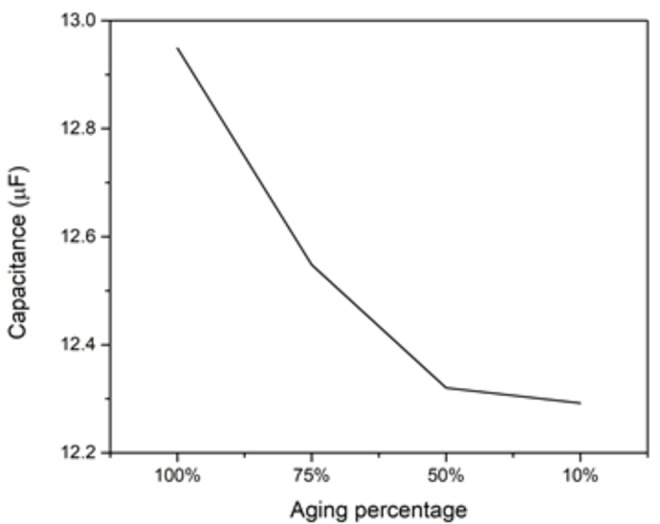
Capacitance vs aging percentage.

**Figure 14:**
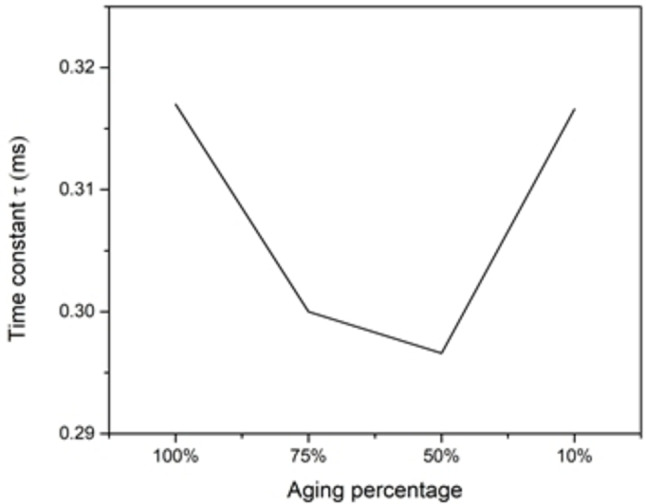
Time constant vs aging percentage.

**Figure 15:**
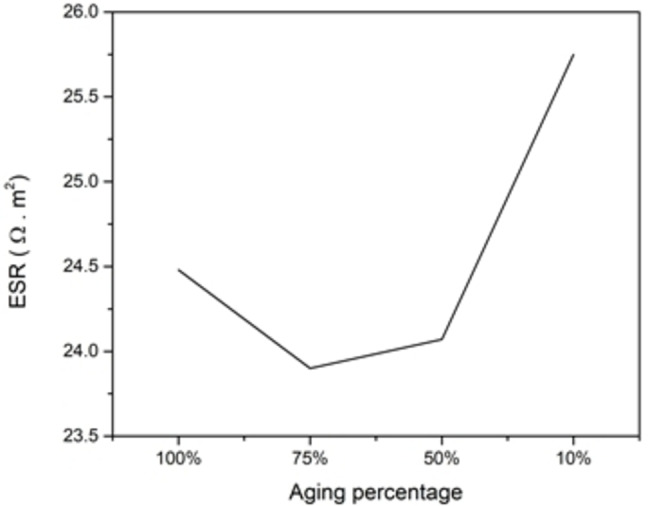
ESR vs aging percentage.

Each time SC charges and discharges it ages, and to capture this effect of aging, the dimensions of the SC changes with time. In this study, aging will be studied through changing the pore cavity opening by decreasing it in percentage steps of its original opening (fully open), while keeping other parameters constant in all simulations. As the opening of the pore decreases, the amount of charges around the electrode that accumulate also decreases (because the surface area of the electrodes shrinks) (Figure 12). This also, leads to capacitance decrement (Figure 13), while the extracted time constant is shown (Figure 14). The ESR values determined from the time constant and capacitance values show (for most part) an increase as the cell ages (Figure 15). Therefore, while the SC ages, its capacitance decreases, and its ESR increases, which is responsible for the efficiency of the SC to deteriorate.

## Conclusions

A parametric study was carried out on a supercapacitor charging kinetics to show the effects of the ionic mobility, solvent material and the pore cavity size of the porous electrode (cell aging). The charging time and the capacitance values was utilized to determine the ESR of the SC and it was found that as the ionic radii decreases (larger atoms), the mobility of the ion species increases and the resulting ESR decreases as in the case of the potassium ions. Also, the use of solvents having a lower dielectric constant (higher conductivity) will give a lower ESR values as shown for the case of acetone. Finally, the decrease in the capacitance values and the increase in the ESR are commonly observed during the aging process of the SC which limits its efficiency. This estimation of ESR can be used in practice to monitor the health of the supercapacitors.

## Conflict of Interests

The authors declare no conflict of interest.

## Data Availability

The data that support the findings of this study are available from the corresponding author upon reasonable request.
